# High-mannose type *N*-glycans with core fucosylation and complex-type *N*-glycans with terminal neuraminic acid residues are unique to porcine islets

**DOI:** 10.1371/journal.pone.0241249

**Published:** 2020-11-10

**Authors:** Yoshihide Nanno, Asif Shajahan, Roberto N. Sonon, Parastoo Azadi, Bernhard J. Hering, Christopher Burlak

**Affiliations:** 1 Department of Surgery, Schulze Diabetes Institute, University of Minnesota, Minneapolis, Minnesota, United States of America; 2 Complex Carbohydrate Research Center, University of Georgia, Athens, GA, United States of America; 3 Safe Food Alliance, Kingsburg, CA, United States of America; Fisheries and Oceans Canada, CANADA

## Abstract

**Objectives:**

Islet transplantation is an emerging treatment option for type 1 diabetes but its application is limited by the shortage of human pancreas donors. Characterization of the *N*- and *O*-glycan surface antigens that vary between human and genetically engineered porcine islet donors could shed light on targets of antibody mediated rejection.

**Methods:**

*N*- and *O*-glycans were isolated from human and adult porcine islets and analyzed using matrix-assisted laser-desorption time-of-flight mass spectrometry (MALDI-TOF-MS) and electrospray ionization mass spectrometry (ESI-MS/MS).

**Results:**

A total of 57 porcine and 34 human *N*-glycans and 21 porcine and 14 human *O*-glycans were detected from cultured islets. Twenty-eight of which were detected only from porcine islets, which include novel xenoantigens such as high-mannose type *N*-glycans with core fucosylation and complex-type *N*-glycans with terminal neuraminic acid residues. Porcine islets have terminal *N*-glycolylneuraminic acid (NeuGc) residue in bi-antennary *N*-glycans and sialyl-Tn *O*-glycans. No galactose-α-1,3-galactose (α-Gal) or Sd^a^ epitope were detected on any of the islets.

**Conclusions:**

These results provide important insights into the potential antigenic differences of *N*- and *O*-glycan profiles between human and porcine islets. Glycan differences may identify novel gene targets for genetic engineering to generate superior porcine islet donors.

## Introduction

Phase 3 trials of transplantation of human pancreatic islets have highlighted the potential of cell replacement therapies in type 1 diabetes [1, 2]. Though highly effective, the applicability of islet transplantation is limited by the shortage of human donor pancreases. Porcine donors are a promising alternative source of islets in light of their similar physiology and size compared with humans, their high reproductive capacity, and the potential for genetic manipulation [3, 4]. Preclinical studies and initial clinical trials of islet xenotransplantation have demonstrated both the safety of porcine islet cell products and the need for developing new and improved startegies for preventing their rejection [5–16].

Carbohydrates (glycans) are one of the major classes of biomolecules found on cell surfaces and play a critical role in biological processes such as organ development and immunity [17]. They are synthesized in the endoplasmic reticulum, modified in the Golgi apparatus, and transferred to the plasma membrane [18]. As glycan expression varies depending on species, strain, individual, organs, and cell types, detailed qualitative and quantitative structural information on the target organ/animal is required. The discovery of the galactose-α-1,3-galactose (α-Gal) epitope that is present in pigs but is absent in humans and nonhuman primates (NHPs) allowed the prevention of hyperacute rejections in pig-to-NHP cardiac and renal xenotransplantation [19, 20]. Recent *in vitro* studies have demonstrated the importance of deleting N-glycolylneuraminic acid (NeuGc) and Sd^a^ antigen on porcine donor cells for preventing rejection of planned solid organ xenotransplants in humans [21–25].

The tissue-specific expression of glycan antigens in primarily avascular islet cell xenografts remains incompletely understood. The α-Gal antigen is expressed only on very few adult porcine islet enocrine cells and does not cause their hypereacute rejection after xenotransplantation in NHPs [7, 26, 27]. To identify potential new targets for genetic engineering of porcine donors tailored for use in islet xenotransplantation, the current study was conducted to compare *N*- and *O*-glycan profiles between human and porcine islets using mass spectrometric analysis. We found unique differences in high-mannose and complex-type *N*-glycan profiles between human and porcine islets, as well as the presence of NeuGc structures in *N*- and *O*-glycans of porcine islets.

## Materials and methods

### Pig and human islet preparation

Seven adult female pigs (*Sus scrofa*; six Mangalista and one Landrace x Yorkshire), with a median age of 3 years (range: 3–4) and a median weight of 206 kg (170–247), were evaluated as donors of islet xenografts. Aliquots of two adult human islet preparations were purchased from PRODO Laboratories Inc. (Aliso Viejo, CA) and one adult human islet preparation deemed unsuitable for clinical transplantation was provided with appropriate consent by our local islet transplant program.

Porcine islets were isolated as previously described [7]. Briefly, donor pig was anesthesized with tiletamine-zolazepam (Telazol^®^; Zoetis, Parsippany-Troy Hills, NJ) before exsanguination, and retrieved pancreas tissue was dissociated with collagenase and neutral protease. Liberated islets were purified from non-islet tissue by continuous density gradient centrifugation on a COBE 2991 cell separator (Terumo BCT, Lakewood, CO), and cultured free-floating in ME 199 media supplemented with 10% heat-inactivated porcine serum (HyClone, Logan, UT), L-glutamine and heparin (10 U/mL) at 37°C in humidified air without CO_2_. From each donor islet preparation, 5,000 islet equivalents of islets were collected and shipped to the Complex Carbohydrate Research Center at the University of Georgia (Athens, GA) in culture media for further analysis. All animal procedures were approved by the University of Minnesota Institutional Animal Care and Use Committee and conducted in compliance with the Animal Welfare Act and adhere to principles stated in the Guide for Care and Use of Laboratory Animals (Protocol Number: 1907-37282A). For the purpose of using human islets, this project was reviewed by the Institutional Review Board of the University of Minnesota and was determined that it does not meet the regulatory definition of human subjects research, as defined by DHHS and FDA and therefore exempt.

### Sample preparation

Islet samples were centrifuged at 400 rcf for 3 min and the pellet was washed five times with 1x PBS, followed by lipid extraction by the Folch method (using chloroform, methanol, and water) [28]. After lipid extraction, the sample was subjected to cold acetone:water precipitation producing a protein-rich powder.

### Release of *N*-glycans

An aliquot of the protein-rich powder was digested with trypsin in Tris-HCl buffer overnight. After protease digestion, the sample was passed through a C18 Sep-Pak cartridge, washed with a 5% acetic acid, and the glycopeptides were eluted with a blend of isopropanol in 5% acetic acid.

The glycopeptide eluate was treated with PNGase F to release the *N*-glycans and the digest was passed through a C18 Sep-Pak cartridge to separate the *N*-glycans from *O*-glycopeptide fraction. The *N*-glycans fraction was eluted first with 5% acetic acid followed by elution of *O*-glycopeptides into another container with a blend of isopropanol and 5% acetic acid. After lyophilization, the *N*-glycans fraction was permethylated for mass spectrometry [29].

### Release of *O*-glycans

The *O*-glycopeptide fraction from each sample was subjected through reductive beta-elimination procedure using sodium borohydride in sodium hydroxide solution to cleave the *O*-linked glycans from the peptides. Subsequently, released *O*-glycans were cleaned up using acid foam (H^+^) ion exchange resin and permethylated for mass spectrometry [29].

### Per-*O*-methylation of *N*-linked glycans

The *N*- and *O*-glycans were permethylated for structural characterization by mass spectrometry [30]. Briefly, the dried eluates were dissolved with dimethylsulfoxide (DMSO) and methylated with NaOH-DMSO base and methyl iodide. The reaction was quenched with water and per-*O*-methylated carbohydrates were extracted with methylene chloride and dried under N_2_.

### Profiling by Matrix-Assisted Laser-Desorption Time-of-Flight Mass Spectrometry (MALDI-TOF-MS)

The permethylated glycans were dissolved with methanol and crystallized with α-dihyroxybenzoic acid (20 mg/mL in 50% methanol: water) matrix. Analysis of glycans present in the samples was performed in the positive ion mode by MALDI-TOF-MS using AB SCIEX TOF/TOF 5800 mass spectrometer (Applied Biosystem/ MDS Analytical Technologies).

### Profiling by Electrospray Ionization Mass Spectrometry (ESI-MS/MS) and Higher-Energy Collisional Dissociation Tandem Mass Spectrometry (HCD-MS/MS)

We conducted ESI-MS/MS to confirm the structure of permethylated glycans. In silico fragmentation of structures predicted based on common mammalian biosynthetic pathway were generated through GlycoWorkbench software. The presence of these fragments was examined on the ESI-MS/MS spectrum of each glycan. If isomers are present or alternate structures are possible, we changed the structural assignments accordingly in order to match the corresponding ESI-MS/MS spectrum.

Aliquot from each permethylated sample glycans was analyzed by ESI-MS/MS (Thermo Orbitrap Fusion Tribrid mass spectrometer) to collect both full mass and MS/MS fragmentation data. Permethylated glycans from the samples were infused into the mass spectrometer through a nano-electrospray ionization (NSI) probe. The MS1 and MS2 spectra (Higher-energy Collisional Dissociation, HCD) of the glycans were acquired at high resolution by a simple precursor scan and total ion monitoring program respectively.

### Data processing of MALDI-TOF-MS, ESI-MS and ESI-MS/MS data

Data Explorer 4.5 (MALDI-TOF-MS data) and XCalibur 4.2 (ESI-MS and ESI-MS/MS data) software were used in order to extract the raw MS data. The quantitation of glycoforms were conducted from MALDI-TOF-MS spectra by calculating the relative intensity of glycan peaks. The glycoworkbench analysis were conducted for the structural assignment of glycans without any further processing of data and by manually entering the values into the software.

## Results

### Characterization of *N*-glycans from pig and human islets

**Fig 1:**
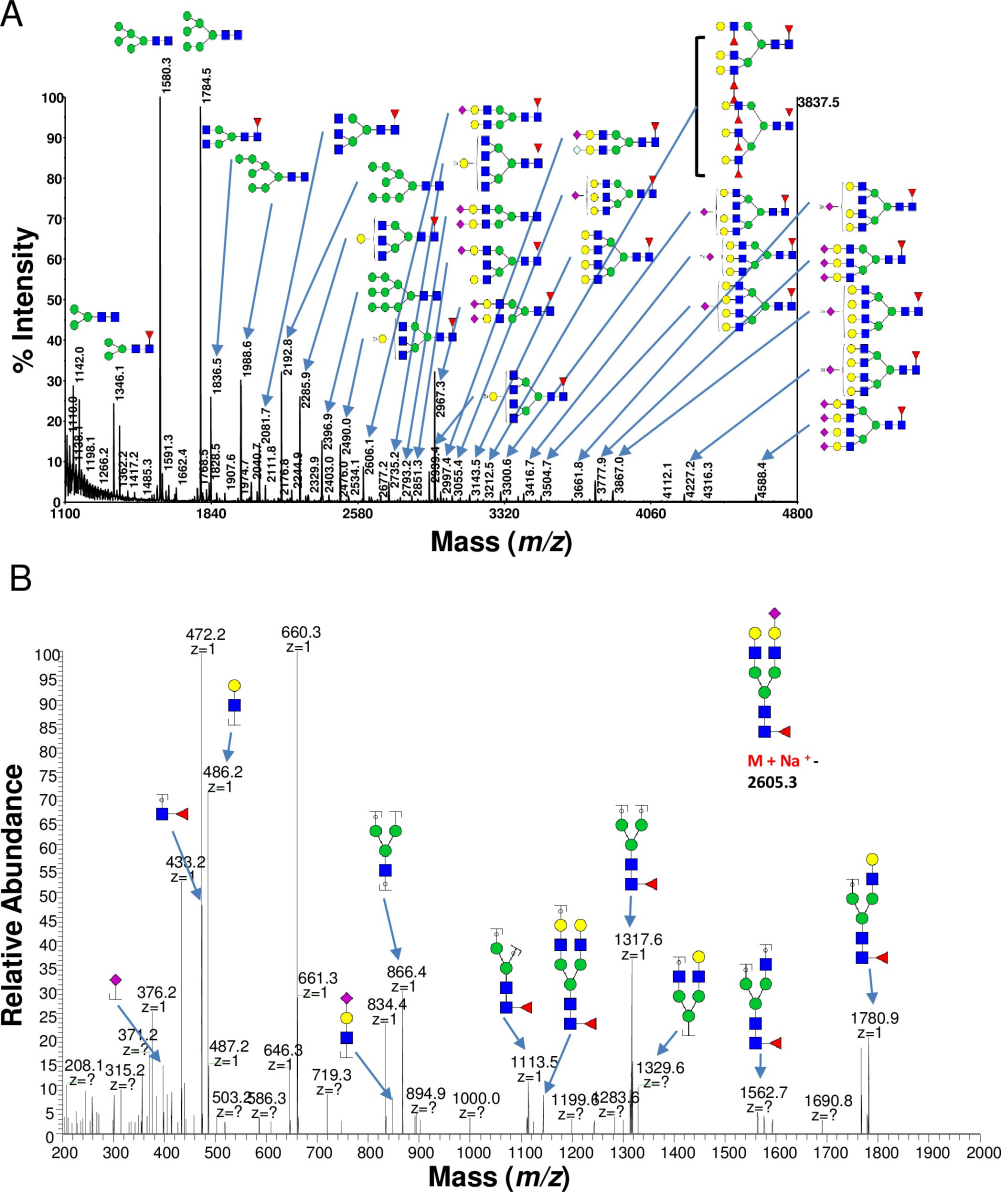
The representative MALDI-TOF-MS and ESI-MS/MS spectrum of *N*-glycans. MALDI-TOF-MS spectrum of permethylated *N*-glycans released by PNGaseF (A) and representative HCD MS2 fragmentation spectra at *m/z* 884 (3+) [M + Na]1+ → 2605.3 (B).

**Fig 2:**
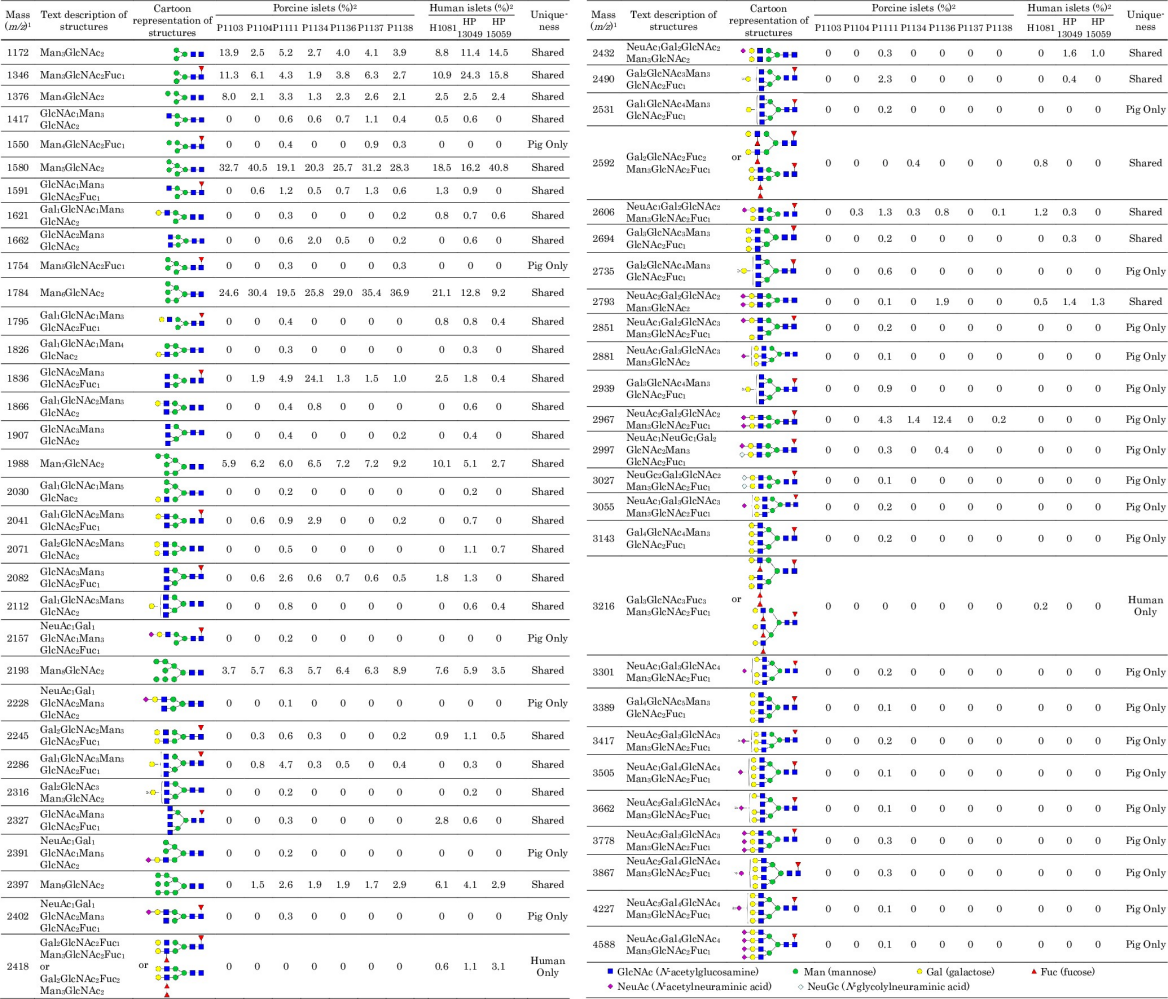
Summary of N-linked glycans detected from adult porcine and human islets by MALDI-TOF-MS and ESI-MS. The structures were confirmed by ESI-MS/MS. ^1^All masses (mass+Na) are permethylated and single-charged values measured by MALDI-TOF-MS. The structure assignment were based on common biosynthetic pathway and ESI-MS/MS fragmentation by HCD. ^2^% *N*-glycans were calculated from the area units of detected *N*-glycans.

**Fig 3:**
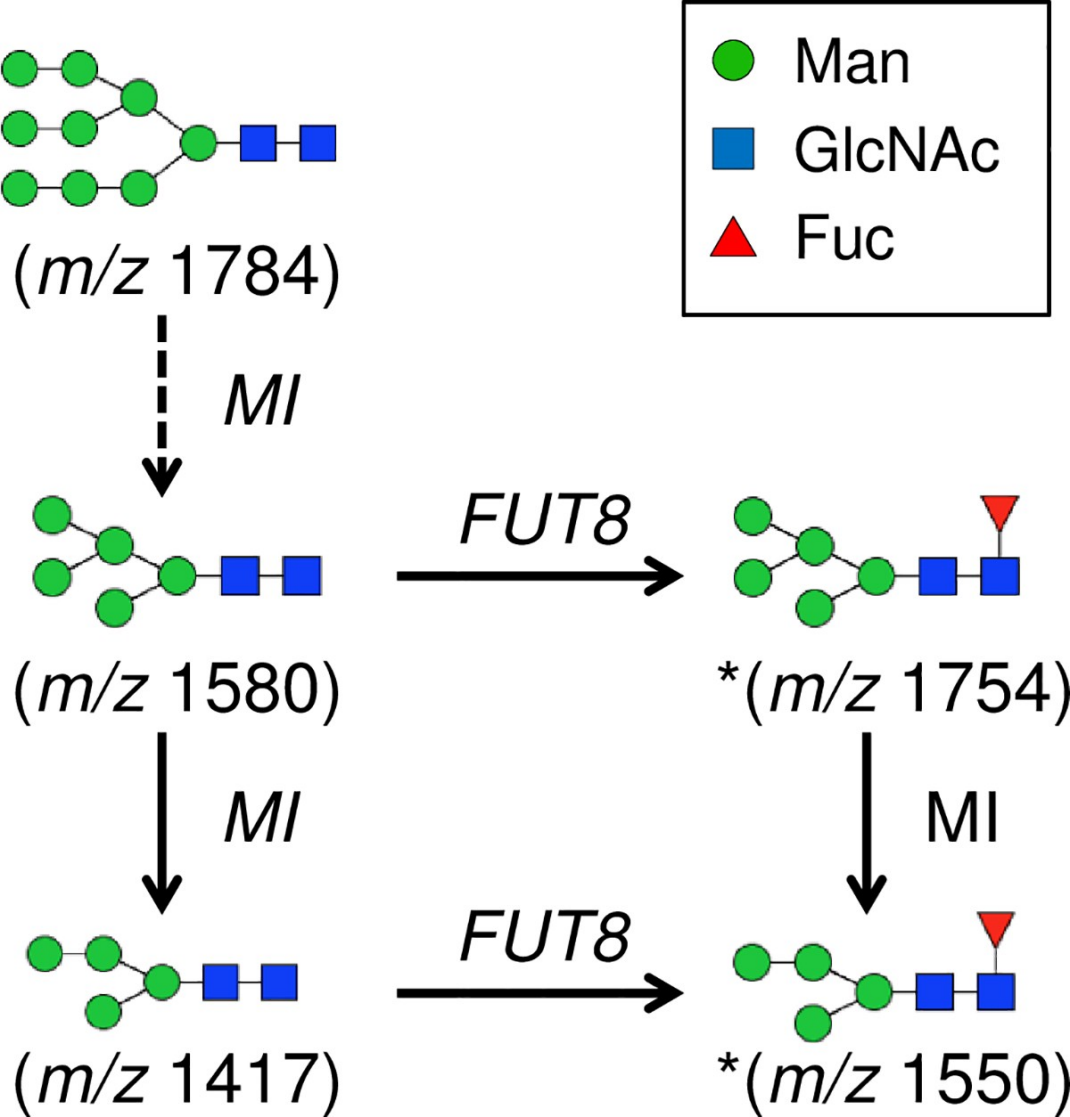
Possible biosynthetic pathways of high-mannose type *N*-glycans with the fucosylated core. FUT8, α1,6-fucosyltransferase; MI, α-mannosidase I.

The representative MALDI-TOF-MS and ESI-MS full scan spectra of the *N*-glycans are shown in Fig 1, and the proposed glycan structures and their percentages are summarized in Fig 2. A total of 57 and 34 *N*-glycans were detected from the porcine and human islets, respectively, 21 of which were only observed from pig islets (Fig 2). High mannose type structures, which included a few fucosylated species, consisted of the majority of the *N*-glycans detected from the glycoprotein. Man5GlcNAc2 (*m/z* 1580) and Man6GlcNAc2 (*m/z* 1784) showed the largest signals of all possible *N*-glycans of porcine islets (Fig 2). Man3GlcNAc2 (*m/z* 1172) and Man3GlcNAc2Fuc1 (*m/z* 1346), as well as Man5GlcNAc2 and Man6GlcNAc2, showed large signals in human islets. There were 2 high mannose type *N*-glycans with core fucosylation (*m/z* 1550 and 1754) annotated in porcine islets, both of which were only found from porcine islets (Fig 3) [31, 32].

**Fig 4:**
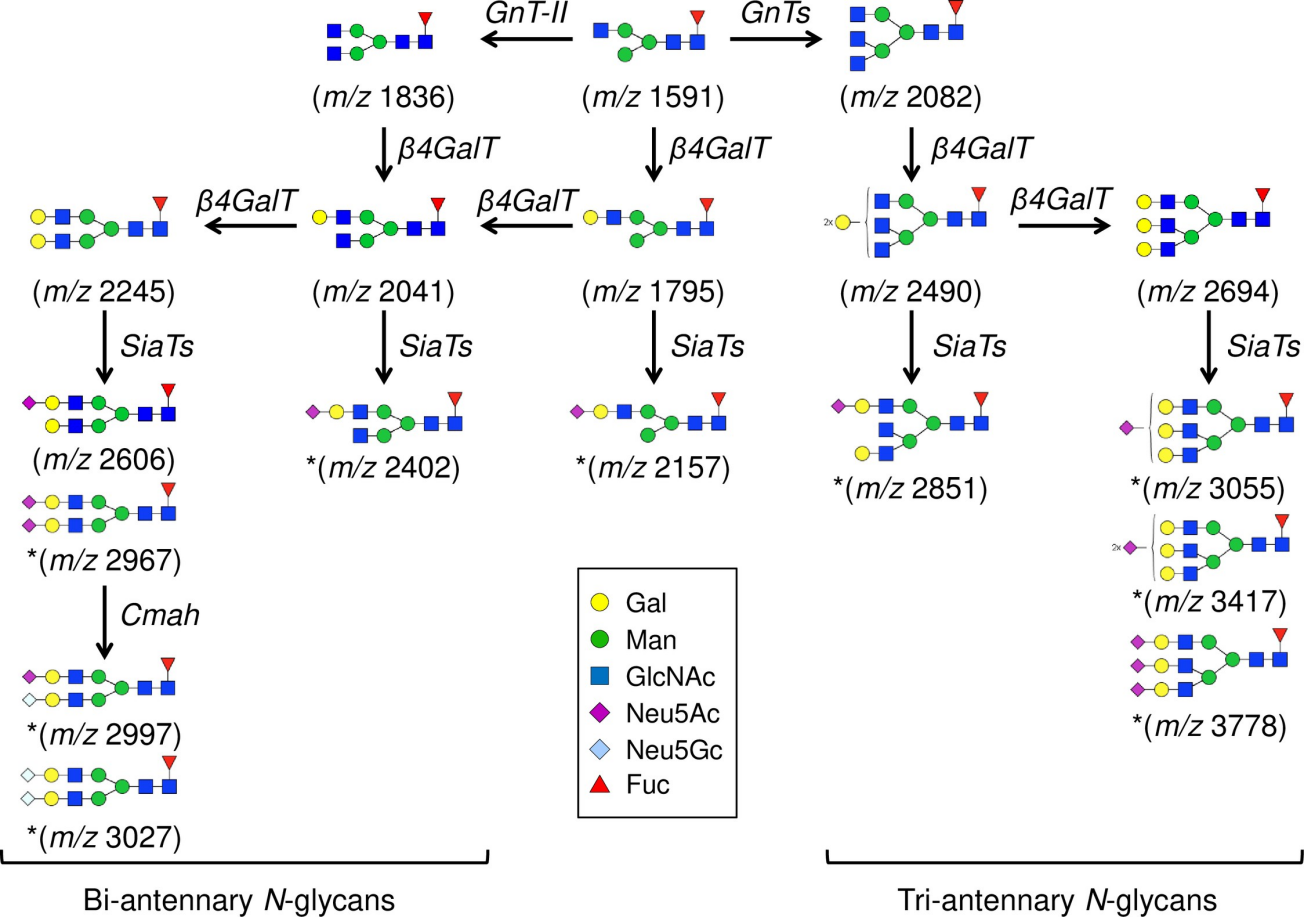
Biosynthetic pathways of bi- and tri-antennary N-glycans. β4GalT, beta-1,4-galactosyltransferase; Cmah, cytidine monophospho-*N*-acetylneuraminic acid hydroxylase; GnT, *N*-Acetylglucosaminyltransferases; SiaTs, Sialyltransferases.

**Fig 5:**
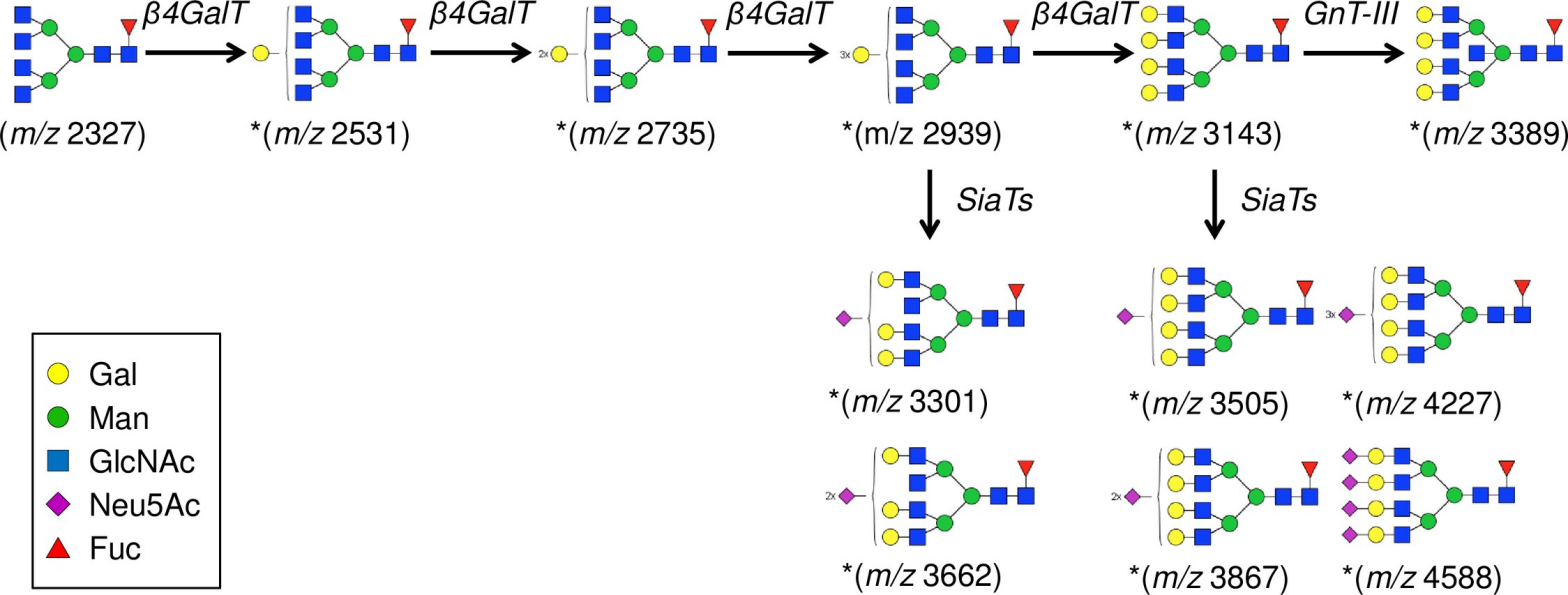
Biosynthetic pathways of tetra-antennary *N*-glycans. β4GalT, beta-1,4-galactosyltransferase; SiaTs, Sialyltransferases.

Approximately 4% of the total *N*-glycans were with terminal neuraminic acid residue, including the two signals (*m/z* 2997 and 3027) that appear to be bi-antennary oligosaccharides contained NeuGc (Fig 4) [33]. Sialylated *N*-glycans in tri- and tetra-antennary structures and hybrid-type structure were only found from porcine islets (Figs 4 and 5) [33]. Moreover, tetra-antennary *N*-glycans with LacNAc elongations (with or without *N*-acetylneuraminic acid [NeuAc] terminal) are only found from porcine islets (Fig 5). Three *N*-glycans with Lewis type structures (*m/z* 2418, 2592, and 3216) were detected from human islets (Fig 2).

### Characterization of *O*-glycans from pig and human islets

**Fig 6:**
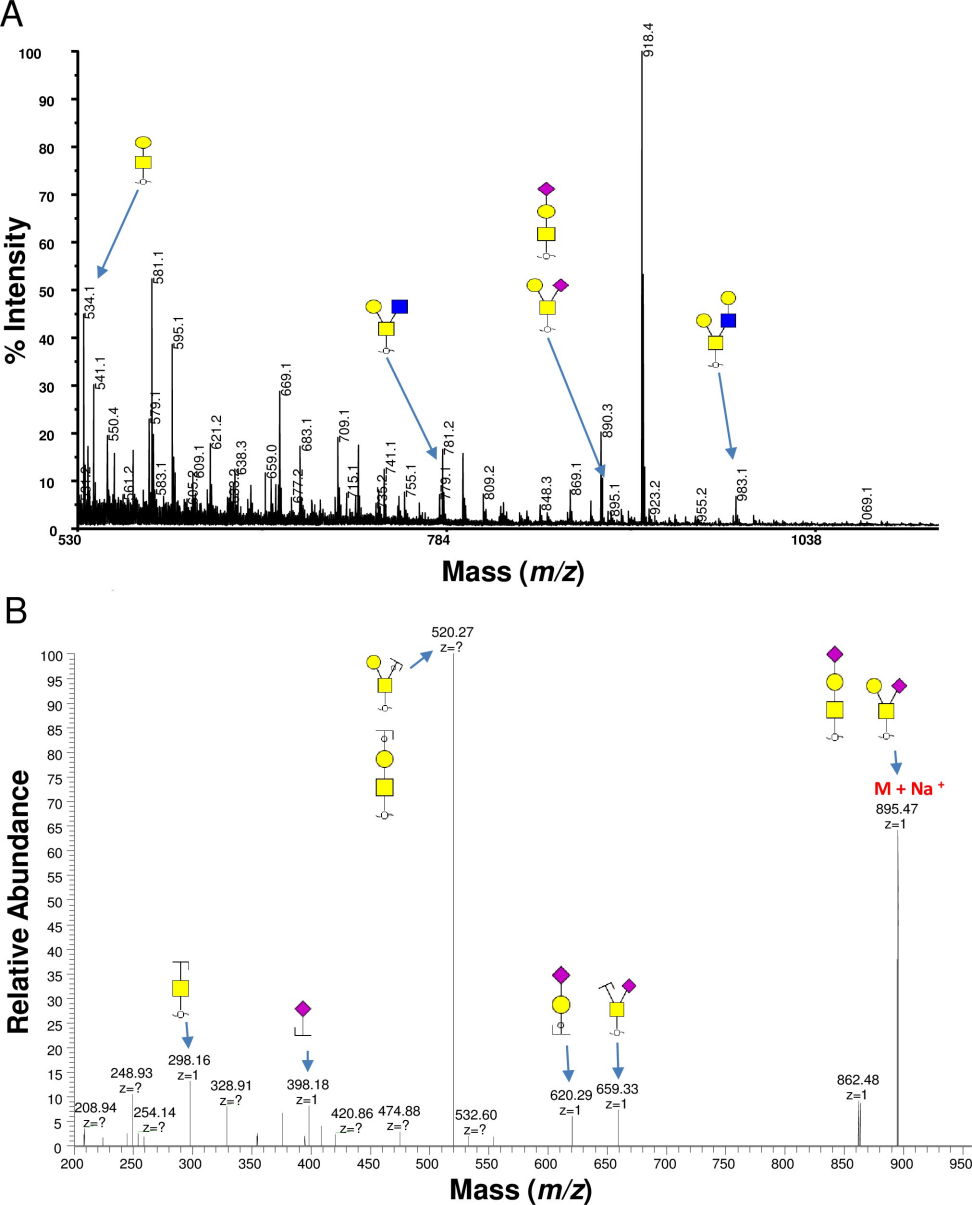
The representative MALDI-TOF-MS and ESI-MS/MS spectrum of *O*-glycans. MALDI-TOF-MS spectrum of permethylated *O*-glycans released by *β*-elimination (A) and representative HCD MS2 fragmentation spectra at *m/z* 895 [M + Na]1+ (B).

**Fig 7:**
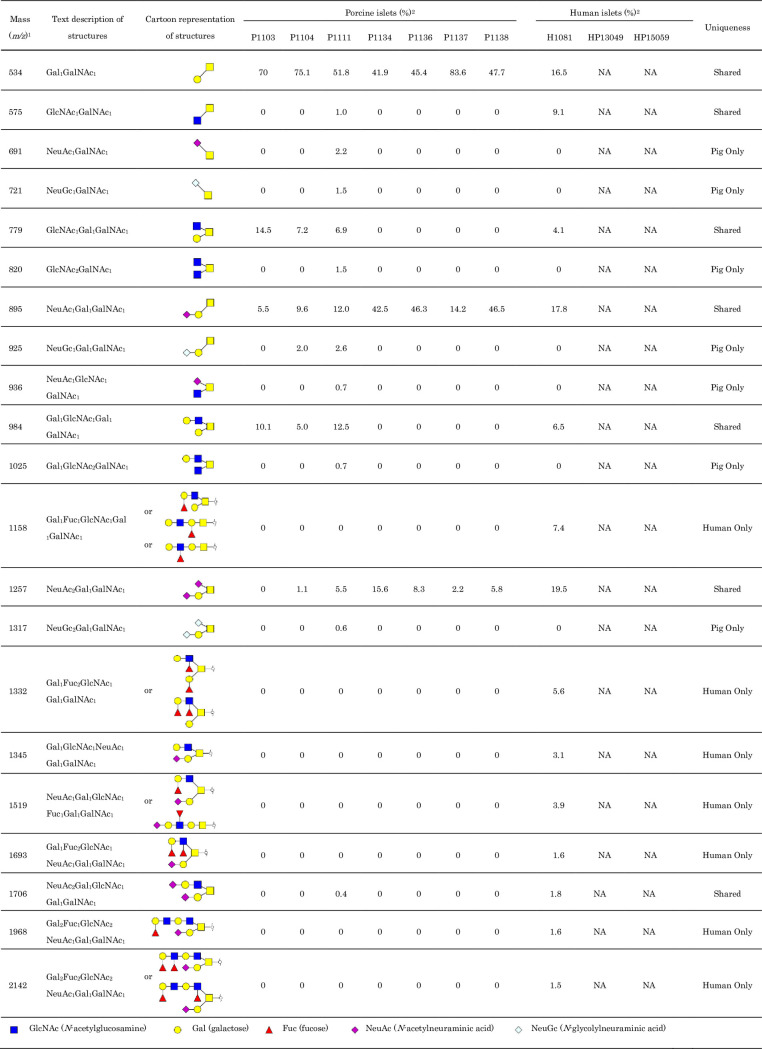
Summary of *O*-linked glycans detected from adult porcine and human islets by MALDI-TOF-MS and ESI-MS/MS. ^1^All masses (mass+Na) are permethylated and single-charged values measured by MALDI-TOF-MS. The structure assignment were based on common biosynthetic pathway and ESI-MS/MS fragmentation by HCD. ^2^% *O*-glycans were calculated from the area units of detected *O*-glycans.

The representative MALDI-TOF-MS and ESI-MS full scan spectra of the *O*-glycans are shown in Fig 6, and the proposed glycan structures and their percentages are summarized in Fig 7. The data from two human islet samples were not obtained due to detergent contamination on the sample which interfered with lower mass *O*-gycan peaks. A total of 21 porcine and 14 human *O*-glycans were detected from islet samples, respectively. Core 1 (*m/z* 534, also known as T antigen) and sialylated core 1 (*m/z* 896) showed the largest signals of all possible *O*-glycans of porcine islets, followed by disialylated core 1 (*m/z* 1257). Most of the *O*-glycans detected from human islets contained the core 1 structure. Three signals that possibly bear the terminal NeuGc residue were observed (*m/z* 722, 926, and 1317) from porcine islets, including sialyl-Tn antigen (*m/z* 722). Lewis type structures such as (Gal)-(FucGlcNAc) and (FucGal)-(FucGlcNAc) were present on the terminal of *O*-glycans of higher mass from sample H1081. To determine the presence of the Sd^a^ antigen, all sialic acid bearing ions from MS/MS spectra were evaluated, however, the presence of the Sd^a^ antigen was not detected.

## Discussion

The demonstration of prolonged diabetes reversal after porcine islet xenotransplantation in NHPs suggests that porcine islets could be developed into a widely available cell source for cell replacement therapy in diabetes. To work toward this end, safer and more effective startegies for preventing islet xenograft rejection will be necessary. Understanding the differences in carbohydrate antigens expressed on procine and human islets could minimize the immunogenicity of islets from porcine donors custom-engineered for use in islet xenotransplantation.

Our MALDI-TOF-MS and ESI-MS/MS analysis of glycans identified differenes between porcine and human islets. Our results confirm that porcine islets have NeuGc in *N*- and *O*-glycan forms, albeit no α-Gal or Sd^a^ epitope was detected from any of the islets (Figs 2 and 7). The study also proposed novel glycan structures that exist only in porcine islets; the high-mannose type *N*-glycans with core fucosylation and complex-type *N*-glycans with terminal neuraminic acid residues, which can be potential gene targets for genetic engineering to generate superior porcine islet donors (Figs 3–5).

Our findings build upon two series of studies that performed qualitative and quantitative analyses of carbohydrate antigens of porcine islets using mass spectrometry (Table 1) [34–38]. Kim *et al*. detected 80 *N*-glycans, which includes NeuGc epitopes and a negligible amount of α-Gal epitope, but they did not include in their analysis a direct comparison between porcine and human islet glycan profiles [34, 35]. Miyagawa *et al*. compared porcine and human islets and reported that 12 of 28 and 9 of 24 *N*-glycans found from adult and neonatal wildtype pigs were detected exclusively on porcine islets, respectively [36, 38]. Within a larger number of 7 porcine donors, we demonstrated 57 *N*-glycans and 21 *O*-glycans including the NeuGc epitope and other potential non-α-Gal antigens, although further studies are needed to confirm these findings as comparison to only 3 and 1 human samples were performed with respect to *N*- and *O*-glycan analysis, respectively. All these studies including ours confirm a negligible amount of α-Gal epitopes and a considerable amount of NeuGc epitopes expressed on adult porcine islets. The number of identified glycans differs between the porcine donors used in our study and donors used in previous studies, which may be attributed to the culture conditions or islet isolation methods. Although direct comparison between the studies may be difficult because of these reasons, the two tri-antennary *N*-glycans (*m/z* 2157 and 2402) were only found in adult porcine islets in ours and Miyagawa’s studies [36].

**Table 1:** Characteristics of mass spectrometry studies for pig islets.

Study	Islet source	Age	# of analysis	Methods	Target	# of detected glycans	α-Gal	NeuGc	Proposed potential non-α-Gal antigens
Kim [34]	SPF CMS miniature pig	N/A	1	MALDI-TOF-MS, ESI-MS/MS, HPLC	*N*-Glycans	80	1^1^	13	N/A
Kim [35]		N/A	1	MALDI-TOF-MS, ESI-MS/MS	GSL	47	0	4	N/A
Miyagawa [36]	WT pigs (Large white/ Landrace x Duroc)	6 months	1^2^	MALDI-TOF-MS, HPLC	*N*-Glycans	28	0	0	Sulfated *N*-glycans
Eguchi [37]		6 months	1^2^	LC-MS	*N*-Glycans	16	0	0	-
Miyagawa [38]		1–3 days	1^2^	MALDI-TOF-MS, HPLC	*N*-Glycans	24	0	0	β-GlcNAc Manα1-3Manα1-6Man
Current study	WT pigs (Mangalista and Landrace x Yorkshire)	3 years	7	MALDI-TOF-MS, ESI-MS/MS	*N*-Glycans *O*-Glycans	57 21	0 0	2 3	Hi-mannose *N*-glycans with core fucosylation Sialylated complex *N*-glycans

Numbers in brackets show reference. CMS, Chicago Medical School; ESI-MS/MS, Electrospray Ionization Mass Spectrometry; GSL, Glycoshpingolipids; HPLC, high-performance liquid chromatography; LC-MS, liquid chromatography mass spectrometry; MALDI-TOF-MS, Matrix-Assisted Laser-Desorption Time-of-Flight Mass Spectrometry; N/A, not available; SPF, specific pathogen-free; WT, wild-type.

^1^detected in ESI-q-TOF MS analysis but the quality was negligible. Zero α-Gal epitope was observed in HPLC analysis.

^2^Islets from several pigs may be combined into one analysis.

An abundance of high-mannose type *N*-glycans is assumed to be a typical feature of islets [36]. In the current study, among the high-mannose type *N*-glycans, a rare type of the high-mannose *N*-glycan that has a fucosylated core (*m/z* 1550 and 1754) were measured from porcine islets. These glycans are previously detected from porcine cathepsin D or lamia bean lectin [39, 40]. Burlak *et al*. has recently described that anti-fucose antibodies in human serum are involved in the antibody-mediated rejection of xenogeneic porcine tissue from donors lacking α-Gal and NeuGc epitopes [23]. It may be reasonable to include high-mannose *N*-glycans with the fucosylated core in future studies to more thoroughly understand xenograft rejection.

In the current study, up to 16% of total *N*-glycans on porcine islets had terminal neuraminic acid residues. Interestingly, all detected sialylated *N*-glycans in tri- and tetra-antennary forms or the hybrid form, which were also detected by Kim *et al*. [34], were only measured from porcine islets but not from human islets. Human islet sialylated *N*-glycans were only found in the bi-antennary form. These findings are in line with the previous study by Komoda et al. that demonstrated that the origin of xenoantigenicity of porcine islets is mainly *N*-glycans including sialic acid antigens (NeuAc and NeuGc) [41].

In addition to the antigens that we discussed above, it has been proposed that Thomsen-Friedenreich antigen (T antigen), T and sialyl-Tn antigens, P antigen, and I or i antigens are potential non-α-Gal carbohydrate antigens against which humans have naturally occurring antibodies [42]. Among these antigens, we successfully observed sialyl-Tn antigens (*m/z* 692 and 721) exclusively in porcine islets (Fig 7), which also can be a target of rejection. To the best of our knowledge, this is the very first study that analyzed *O*-glycan structures of pig and human islets. However, as mentioned above, the condition of the islet isolation and culture might affect the result of the analysis and further studies are warranted to confirm these findings.

In this study, islets from 3-year old adult pigs were examined for *N*- and *O*-glycan analyses building on studies of glycan expression previously reported by age [43] Future studies should examine the glycan expression of neonatal porcine islets to describe alternative age donors for clinital transplantation.

In conclusion, evidence presented in this study indicates that core-fucosylated high-mannose *N*-glycans and complex-type *N*-glycans with terminal neuraminic acid residue are unique structures found on porcine islets. Also, the structural analysis suggested that NeuGc structures are found as a part of mature bi-antennary *N*-glycans or sialylated Tn antigens in *O*-glycans. This study underscores the opportunities associated with improved understanding of specific *N*- and *O*-glycans expressed on porcine islets. Future studies will focus on the antigen-specific reactions against these novel glycan structures, which can be a potential target of xenoreactive antibodies.
